# A Survey on Recent Advances in Wearable Fall Detection Systems

**DOI:** 10.1155/2020/2167160

**Published:** 2020-01-13

**Authors:** Anita Ramachandran, Anupama Karuppiah

**Affiliations:** ^1^Department of Computer Science & Information Systems, BITS, Pilani, Bangalore, India; ^2^Deptartment of Electrical & Electronics Engineering, BITS, Pilani, KK Birla Goa Campus, Goa, India

## Abstract

With advances in medicine and healthcare systems, the average life expectancy of human beings has increased to more than 80 yrs. As a result, the demographic old-age dependency ratio (people aged 65 or above relative to those aged 15–64) is expected to increase, by 2060, from ∼28% to ∼50% in the European Union and from ∼33% to ∼45% in Asia (Ageing Report European Economy, 2015). Therefore, the percentage of people who need additional care is also expected to increase. For instance, per studies conducted by the National Program for Health Care of the Elderly (NPHCE), elderly population in India will increase to 12% of the national population by 2025 with 8%–10% requiring utmost care. Geriatric healthcare has gained a lot of prominence in recent years, with specific focus on fall detection systems (FDSs) because of their impact on public lives. According to a World Health Organization report, the frequency of falls increases with increase in age and frailty. Older people living in nursing homes fall more often than those living in the community and 40% of them experience recurrent falls (World Health Organization, 2007). Machine learning (ML) has found its application in geriatric healthcare systems, especially in FDSs. In this paper, we examine the requirements of a typical FDS. Then we present a survey of the recent work in the area of fall detection systems, with focus on the application of machine learning. We also analyze the challenges in FDS systems based on the literature survey.

## 1. Introduction

Intelligent IoT-based ambient assisted living systems (AALS) for the elderly have been a major research focus area in recent times. According to the studies conducted by National Program for Health Care of the Elderly (NPHCE), elderly population in India will increase to 12% of the national population by 2025 with 8%–10% requiring utmost care. Application of machine learning in areas of AALS such as fall detection, therefore, has the potential to have a huge public impact. Much work has been done in the area of fall detection systems and the application of machine learning to such systems to enable fall classification, detection, and prediction. We have been working on the development of an FDS which applies the biological profile of a subject to classify him into a risk category pertaining to his fall probability. The three categories we have defined are high risk, medium risk, and low risk. The categorization thus derived, along with parameters from a wearable sensor, is then applied to ML algorithms to detect falls. The objective of this paper is to bring out an extensive literature survey of the recent work in the area of fall detection systems, with focus on the application of machine learning to wearable sensor-based approaches.

The rest of the paper is organized as follows. First, we examine the desirable requirements of a wearable fall detection system. Then we present an overview of FDSs based on environmental sensors, vision-based systems, and wearable sensors. Subsequently, we dwell a little deeper into the recent advances in FDS based on wearable devices. In this, we present a literature survey on threshold-based mechanisms and machine learning-based algorithms in fall classification and detection. It was observed that different papers examined different performance parameters of ML algorithms, and we present a summary of the results here. Lastly, existing literature shows that various biological, physiological, and environmental parameters affect a subject's risk of falling. We present results from our study on the various biological factors that impact the probability of fall in elderly persons. We conclude our paper with the challenges we observed in the existing fall detection solutions.

## 2. Requirements for Fall Detection Systems

The purpose of FDSs is automatic detection of falls and enabling of assistance by caregivers when required. FDSs primarily find its application in geriatric care because falls are more frequent and severe in the elderly. Because such systems would be used by the elderly, it is important for the manner in which falls are detected to be nonintrusive. For example, a wearable sensor that is heavy or causes inconvenience to the subject may not be a popular solution.

Power consumption of the apparatus should be minimized because there is a possibility of the subject forgetting to charge it. This entails that the sensors and the network design of the system have to be optimized for power consumption.

An FDS should not restrict the subjects' mobility, and they should be free to move around the area they want to. Camera-based or infrared- (IR-) based systems [3] may restrict the subject to be within a certain region of interest (ROI), while wearable sensor-based systems do provide more mobility to the subjects. An IR- or camera-based system may employ multiple techniques to increase the coverage, such as sweep coverage-based deployments, but this would mean increasing the cost of the system.

Another important requirement of an FDS is that it should be able to distinguish between falls and activities of daily life (ADLs) or near-fall conditions. This is to prevent triggering of premature action in the case of false positives and adequate care not being provided in the case of false negatives. Therefore, the accuracy of detection is an important factor.

The manner in which triggers are generated on fall detection is also important. Caregivers may be located remotely, and an FDS should support providing notifications to remote personnel. The notification could be a short indication like a message or could be a descriptive account from the ROI, by way of images captured by a camera. The former is easier to implement, while the latter has the advantage that it can provide a clear picture of the impact to the observer before deciding a course of action.

Latency is another factor that should be considered in the design of FDSs. Delay in the detection of falls and that between the detection of falls and notification of the caregiver should be minimized for the FDS to be effective. This implies that techniques used for fall detection should be delay-sensitive. It also implies that the network design should provide high quality of service for data packets that are generated as a result of fall detection, in comparison with messages for keepalive or periodic reporting of sensor readings.

It is also desirable for an FDS to keep track of a subject's biological parameters and fall history so as to have the capability of predicting falls before their occurrence. This would involve reporting of the biological parameters by the sensor nodes periodically and application of data analytics and machine learning techniques on the data collected over a period of time.

## 3. Fall Detection Systems: An Overview

The current research on the wearable system design for a geriatric healthcare monitoring system for detecting falls can be broadly classified as follows:

- Environmental sensing-based systems
- Wearable sensor-based systems
- Vision-based systems

Environmental sensing-based systems work on input from sensors placed in the environment. Some examples of such systems are infrared sensor and acoustic and passive infrared (PIR) motion detector-based systems. Infrared sensor-based systems sense certain characteristics of an ROI to detect a fall, either by emitting or detecting infrared radiation or by measuring the heat being emitted by an object and detecting motion. Microphone-based FDSs use acoustic signals to detect sound in a room, locate the source, and classify it as a fall or a nonfall condition. Motion detector-based systems identify falls based on detection of motion within an ROI. As an example of environmental sensing-based systems, Taramasco et al. [4] explain one which uses very-low-resolution thermal sensors located on two horizontal planes along the floor, for classifying falls. The algorithms compared were three recurrent neural networks (RNN)—long short-term memory (LSTM), gated recurrent unit, and Bi-LSTM, with Bi-LSTM giving an accuracy of 93%. In [5], the authors rely on the existing wireless infrastructure for detecting falls. The channel state information (CSI) from WiFi deployments in a given area was used for this purpose. Support vector machines (SVMs) and Random Forest algorithms were applied to CSI matrix for device-free fall detection. The experiments were conducted in controlled environments with falls simulated by one subject at a time. The advantages of this system are that it is nonintrusive, which means it does not expect the subjects being monitored to be wearing or carrying any device. However, it may be high on false positives because of the impact of ambient parameters such as heat; for example, since the system relies on thermal sensors, its accuracy is affected by the presence of other exothermic devices such as heaters. This study also observed that when multiple people are in the region under experimentation, their combined movements influenced the accuracy of the outcome. Also, in this paper, false positives, though at acceptable levels, were high, because of the small dataset used for training and testing. Ciabattoni et al. [6] describe a system consisting of a low-cost mobile robot and an RGB camera, deployed in a room, that produces real-time video stream. The robot employs deep learning techniques for user detection, position estimation to detect a fall, photo and video capture, and interfacing with a Bot telegram. The accuracy reported for fall detection was 93%.

Vision-based systems do not perform any parameter monitoring of the subjects; instead, they rely on image processing techniques on the video frames or images captured by cameras around the ROI. ML algorithms may be applied over image processing techniques to enable more accurate fall detection. In [7], convolutional neural networks (CNNs) are trained on different datasets of optical flow images. This helps the network to detect different actions. Transfer learning is then applied from action recognition to fall detection. The experiment was conducted on 3 different datasets and reported an accuracy of above 95% in all cases. However, one stated drawback of this approach is that it is susceptible to inaccuracies resulting from ambient lighting changes. Zerrouki et al. [8] detail a comparative study of ML algorithms for fall detection with video sequences during different daily and fall activities as input. They compared Naïve-Bayes, k-nearest neighbors (kNN), neural network, and SVM algorithms and concluded that SVM performed best among these, with respect to accuracy, sensitivity, specificity, precision, recall, F-measure, and area under the curve (AUC). Anishchenko [9] applies deep learning and transfer learning techniques on data generated by surveillance cameras under realistic conditions, to detect falls. The objective was to overcome the setbacks of simulated datasets collected under controlled environments. Bhandari et al. [10] analyze video frames for fall detection in 3 steps—finding out interest points using Shi-Tomasi algorithm, calculating the distance between interest points from optical flow calculation with Lucas–Kanade algorithm, and estimating the speed and direction of motion to conclude whether a fall has taken place or not. The method is another example of application of unsupervised learning in fall detection. The accuracy reported is 95% for nonfall activities and 96.67% for fall activities. In [11], data were collected using a Kinect camera and a triaxial accelerometer. The video input was used to classify the accelerometer data into falls or nonfalls, in the training phase. Time and frequency domain analysis was performed on the data—the former using SVM and the latter using lifting wavelet transform. It is reported that the frequency-based analysis exhibited an accuracy of 100% in detecting falls, while the SVM-based time domain analysis reported 98.31%. Yanfei et al. [12] analyze feeds from a Kinect camera and processes point cloud images to detect falls and reduce false positives. More recently, the application of deep learning techniques to fall detection has become an active area of research. Lu et al. [13] use video feeds from ambient data and applies CNN and LSTM for feature extraction. The use of 3D CNN enables extraction of motion features from temporal sequence, in addition to spatial information, while LSTM-based visual attention mechanism is used to locate the regions of interest. The authors note that this approach works well on small datasets and that analysis of long motion sequences using this scheme will increase the computational costs of the system. While vision-based systems provide accurate details of abnormal conditions to a remote caregiver via images or video feeds, they tend to be more expensive and computationally intensive and require higher processing time, in addition to being a subtle intrusion to the privacy of the subjects.

In wearable device-based FDSs, the sensors used for fall detection are embedded within a wearable device worn by the subject, such as a wrist band. The parameters monitored by such systems include the following: heart rate variability (HRV), electrocardiogram (ECG), pulse oximetry (SPO_2_), and kinematic attributes measured by accelerometers, gyroscope, and magnetometer. The data reported by wearable sensors are fed as inputs to a threshold-based system or as feature sets to a machine learning-based system to classify and detect falls. Wearable sensor-based systems are less expensive, have low power consumption which reduces the overheard on charging the system, and are usually in the form of a band that can be worn around the wrist or thigh, which is less susceptible to being separated from the subjects. Kaewkannate and Kim [14] provide a summary of comparison between four wearable wrist-band style devices currently available on the market with respect to their features and cost. The power consumption of wearable devices is dependent on the device configuration, type of sensors, and communication technologies used. Oletic and Bilas [15] give an analysis of total power consumptions for different operating scenarios, for certain configurations of wearable devices. There are also systems where the sensors are worn not on the wrist but on other parts of the body, or are embedded within a smartphone. Smartphone-based systems expect the subject to charge their device as required and carry it with them to enable fall detection, which are not good prerequisites for geriatric healthcare systems.

There have been some papers that propose an end-to-end IoT-based system for fall detection. An example of such a system in indoor environments is presented in [16]. This design makes use of low-power wireless sensor networks, smart devices, big data, and cloud computing. A 3D-axis accelerometer embedded into a 6LowPAN device wearable collects movement information and applies decision tree algorithms to detect falls.

## 4. Relevance of Machine Learning in Fall Detection

**Figure 1:**
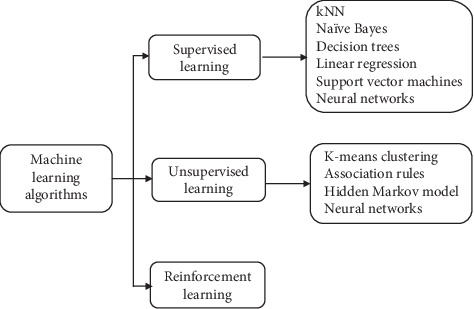
Taxonomy of machine learning algorithms.

Machine learning is a technique that applies mathematical models on datasets to analyze, classify, and discover new meanings from them, to enable the system to learn automatically from the training it received. A model trained on a given dataset is capable of interpreting new input data and predicting outcome variables. Machine learning helps achieve certain amount of task and decision automation for various domains. There are 3 types of machine learning approaches—supervised machine learning, unsupervised machine learning, and reinforcement learning. In supervised machine learning, the training is done based on labelled input data. For every input data, there is a corresponding outcome variable. Therefore, the input data is classified a priori, and when there is a new data point, it can be mapped to one of the defined classes. There are two approaches to supervised machine learning—regression and classification. Unsupervised learning is when the algorithm itself tries to find a pattern within a given dataset. Reinforcement learning allows the system to adapt its behaviour based on feedback or rewards from the environment [17]. In each of the categories of machine learning approaches, there are multiple algorithms. A simplified taxonomy of machine learning algorithms is given in Figure 1.

Due to the advances in the field of medicine and changes in population demographics, geriatric healthcare has gained a lot of significance. In the recent years, there has been widespread application of technology in the healthcare domain. Some applications of machine learning in geriatric healthcare include monitoring of vitals, analysis of sleep patterns, behavioural studies, and fall detection—the fundamental objective of these applications being to detect and/or predict abnormalities. Machine learning in fall detection helps in intelligently detecting falls based on a subject's activity patterns. It may be easy for a fall detection system to raise an alarm whenever a change in activity pattern is observed; however, this would result in excessive alarms being triggered falsely. If the fall detection system is designed to be conservative in raising alarms, then it may not raise alarms when actual falls occur. Hence, it becomes important that false positives and false negatives in a fall detection system are minimized, and the self-learning capability of machine learning algorithms plays a vital role here. Other performance parameters of machine learning algorithms include specificity, sensitivity, and recall. Algorithms for fall detection work on datasets generated by camera, environmental sensors, or wearable sensors, and the objective of research in this area is to improve the performance parameters of the algorithms when applied to fall detection.

**Figure 2:**
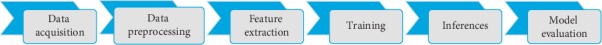
Flow diagram for machine learning-based model building.

In the context of fall detection, the outcome variables of machine learning algorithms for binary classification would be falls or ADLs. ADLs include various postures such as sitting, standing, lying down, and slow or fast transitions between these activities. In order to ensure that the outcome variables predicted by a machine learning system are correct, data cleaning and preprocessing are performed on the fall dataset. Subsequently, feature extraction is performed so as to shortlist the right set of features that will characterize the dataset, and this set of features is used for creating a trained model (see Figure 2). For research in machine learning-based fall detection systems, new datasets for falls and ADLs are created by experimentations in controlled environments, or publicly available datasets are used for analyses. Apart from the direct application of machine learning algorithms to such datasets to detect a fall or an ADL, existing literature also shows various techniques for feature extraction and nullifying the errors induced by external factors such as misplacement of sensors.

## 5. Fall Detection Using Wearable Sensors

There are two approaches to fall detection using wearable sensors—threshold-based systems and machine learning-based systems.

### 5.1. Threshold-Based Wearable Fall Detection Systems

Threshold-based systems have been a widely researched area. The focus of such research has been on multiple aspects, such as ability to detect falls and classify falls from ADLs and near-fall conditions and sensor fusion of readings from multiple sensor nodes.

In [18], an algorithm based on first differences and first derivatives of sum of accelerometer readings along *X*, *Y*, and *Z* directions is described. This algorithm is real-time and reliable and was capable of distinguishing jerky movements from falls. Wu et al. [19] build a system with triaxial accelerometer and proposes an algorithm based on thresholds of sum acceleration and rotation angle information. This combines threshold values of acceleration with quaternion rotation, to conclude whether a fall has taken place or not. The sensitivity and specificity of this algorithm are reported to be better than pure threshold-based systems.

In systems where only accelerometer is used, the accuracy of threshold-based fall detection may not hold true in all conditions. Sensor fusion techniques have been experimented in some cases, where sensors other than accelerometers have been applied. For example, in [20], the author makes use of an accelerometer combined with an HRV sensor. The signals from the accelerometer are analyzed for abnormalities in movements. The signals from HRV sensor are analyzed for abnormalities in heart rates induced by anxiety at the time of fall. Both the analyses are threshold-based and performed independently, and a fall is concluded to have occurred if both report the occurrence of a fall. The accuracy of the ability to distinguish falls from identical activities in this study was reported to be between 96% and 100%. In [21], a three-step algorithm is proposed based on activity intensity analysis, posture analysis, and transition analysis, with signals reported by accelerometer and gyroscope. Results show sensitivity of 91% and specificity of 92%, in being able to separate falls from ADLs and near-fall conditions. In addition to the application of ML algorithms to multiple wearable sensor node readings, there have also been experiments on building context around the sensor readings from the surroundings. In [22], the authors consider acceleration, pulse, and oxygen saturation of the subject via an Android phone, combined with context awareness being incorporated by PIR motion, door contact, pressure mats, and power usage detectors. Sensor fusion among these disparate sources is achieved by Bayesian networks to perform fall detection.

Chen et al. [23] use microelectromechanical system (MEMS) accelerometers for fall detection. It says that in the experiments that the authors conducted, setting thresholds separately for the 3 axes did not work well. Hence, the norm of the 3 axes was taken, and a threshold was set for the norm. The authors note that there is scope for improvement in performance if the design is customized, since the acceleration profiles vary from person to person depending on his physique.

Tsinganos Skodras [24] compiles various sensor fusion techniques applied for fall detection and also summarizes their performance results in the context of fall detection. In most cases highlighted in this study, the sensors used were accelerometers and gyroscopes. In our research too, we find that although there are cases where multiple sensors are used, most of the research studies use only IMU-based sensors. The lack of application of threshold-based mechanisms with sensor fusion techniques could be because of the limited capabilities that a pure threshold-based system presents in decision making under dynamic uncertainties.

Threshold-based algorithms are typically designed to minimize computational overhead. However, the threshold values may vary with the placement of sensors and individual activity patterns. In [25], the authors state that the thresholds and positioning of sensors impact the accuracy of fall detection. They conducted experiments by placing the sensors on the shoulder, waist, and foot of the subjects. A series of observations were made by adjusting both the thresholds of acceleration for fall detection and the placement of sensors for improving the performance of the system. Sensor placement on the waist resulted in lesser false positives than that on the shoulder and foot.

In order not to rely only on numerical thresholds set on acceleration, Kostopoulos et al. [26] consider the subject's rebound and residual movements in the postfall phase for fall detection, in addition to a threshold-based analysis of accelerometer data. The maximum and minimum thresholds of acceleration over a short duration of time are used to determine occurrence of a fall. The rebound is calculated as the difference between this max and min thresholds. The classification of fall is done subsequently, again based on the acceleration values. The postfall analysis is used to determine the impact of the fall, which in turn decides whether an alarm raised to the caretaker has to be cancelled or not.

There are also systems that have been proposed which work based on sensors embedded in mobile phones. A disadvantage with such systems is that the subject should remember to charge and carry mobile phones as a prerequisite for the system to detect falls. Another drawback is that not all mobile phones may come equipped with the required sensors. There is also the possibility of false positives caused by mobile phone drops. Chaitep and Chawachat [27] proposed a threshold-based detection method which makes use of G-force values derived from accelerometer readings to identify falls and smartphone drops. The algorithm consists of 3 phases—checking for a smartphone drop, detecting a fall, and fall confirmation.

A snapshot of recent research on fall detection using threshold-based wearable systems is presented in Table 1. In threshold-based systems, a maximum value for the parameters read by sensors such as an accelerometer or gyroscope is predefined. A measured value beyond this threshold is an indicator of an abnormal event. Such systems are simple to implement and are computationally less intensive. However, there are some drawbacks in such system, as detailed above. Setting a wrong threshold may lead to lower accuracies in fall detection. Also, the thresholds themselves may be different across subjects because of differences in their ADL patterns. The alternative machine learning-based approaches use supervised or unsupervised algorithms on large datasets to train classifiers and thus build the ability to recognize a fall. The model thus trained can be used to detect falls for other input data. This is described below.

### 5.2. Machine Learning-Based Wearable Systems for Fall Detection

While threshold-based systems have been popular because of its low computational overhead, it could be prone to more false positives and false negatives, given that the thresholds themselves may be affected by various factors. As a result, machine learning algorithms for fall detection have been a much researched area. There has been extensive research into the efficiencies of various machine learning techniques for fall detection. For example, de Quadros et al. [28] compare threshold-based mechanism and machine learning-based mechanisms for fall detection applied on data generated by accelerometer, gyroscope, and magnetometer. The paper concludes that the machine learning-based mechanism yielded much better results than the threshold-based solutions.

Machine learning-based techniques differ from each other in multiple factors—the feature set used, sensors employed, placement of sensors, algorithms applied, dataset used, performance parameters monitored, and so on. There are studies that focus primarily on the application of supervised learning suited to fall detection using wearable sensors with good performance results. Özdemir and Barshan [29] take into account features of acceleration, rate of turn, and the strength of the Earth's magnetic field along three perpendicular axes to detect a fall. The algorithm used distinguishes falls from ADLs using six machine learning techniques: kNN, least squares method (LSM), SVM, Bayesian decision making (BDM), dynamic time warping (DTW), and artificial neural networks (ANNs). The factors monitored for performance comparison are sensitivity, specificity, accuracy, true positives, true negatives, false positives, and false negatives. The observation was that kNN and LSM methods do not miss any falls and hence were concluded as reliable classifiers. Choi et al. [30] compare ML algorithms for fall detection using a single node and two nodes. The results reported an accuracy of 99.4% for classification, with single node consisting of a 3-axis accelerometer and a 2-axis gyroscope, worn at the chest level. With 2 nodes, a second node with a 3-axis accelerometer and a 1-axis gyroscope was worn on the thigh, in addition to the first node worn at the chest. The accuracy in this case was 99.8%. Naïve-Bayes classifier gave the best results in both cases. In [31], the dataset used was generated from accelerometer and gyroscope, placed at the waist level. Feature extraction was performed using windowing technique, feature selection using rank-based system, and classification using Naïve-Bayes, LSM, ANN, SVM, and kNN algorithms. kNN, ANN, and SVM had the best performance results compared to LSM and Naïve-Bayes. Results show an accuracy of 87.5%, sensitivity of 90.70%, and specificity of 83.78%, for kNN. Jefiza et al. [32] use backpropagation neural network (BPNN) for fall detection, with data collected from 3-axis accelerometer and gyroscope, and reported an accuracy of 98.182%, precision of 98.33%, sensitivity of 95.161%, and specificity of 99.367%. Hossain et al. [33] also attempt to distinguish falls from ADLs and compares SVM, kNN, and complex tree algorithms applied on data generated by accelerometers. The paper compared the performance of these algorithms with respect to accuracy, precision, and recall, on ADLs and four types of falls (forward, backward, right, and left). It was observed that the accuracy and precision of SVM were the highest, while complex tree performed better in terms of recall analysis. Machine learning algorithms, much like threshold-based techniques, have also been applied to sensors integrated with mobile phones. In [34], a method for fall detection an classification by machine learning using mobile phones is proposed; the features used were acceleration and the algorithms compared were SVM, sparse multinomial logistic regression (SMLR), kNN, decision trees, and Naïve-Bayes. Results showed that both SVM and SMLR were able to identify a fall with 98% accuracy and classify the type of fall with 99% accuracy.

Despite supervised learning techniques finding more application in fall detection, as detailed above, the application of unsupervised learning is also not uncommon. In fact, Lee et al. [35] claim that supervised learning has deficiencies in terms of abnormality detection and activity classification. The authors hence experimented with unsupervised learning for fall detection. Their algorithm creates an activity probability model of a subject's past activity information from accelerometer readings. This model is then used to determine whether an activity is abnormal or not. The advantage of this approach is that it achieves a certain level of personalization in fall detection since the probability density function which is central to activity comparison is developed per subject.

One of the observed drawbacks of wearable sensors is that the accuracy of fall classification and detection is impacted by the placement of the sensors. In [36], the authors generated a dataset with accelerometer and gyroscope, worn around the waist, and applied SVM, boosted and bagged decision trees, kNN, k-mean, and hidden Markov model (HMM). It was observed that fine kNN produced an accuracy of 99.4%. Yu et al. [37] attempt to reduce errors caused by incorrect sensor positions and details an HMM-based fall detection system for the same. Sensor orientation calibrations are applied on HMM classifiers to resolve issues arising out of misplaced sensor (3-axis accelerometer) locations and misaligned sensor orientations. This paper reports sensitivity of 99.2% on an experimental dataset, 100% for a real-world fall dataset.

Guvensan et al. [38] focus on energy efficiency in fall detection. A combination of threshold-based method and ML-based algorithms—K-Star, Naïve-Bayes, and J48—was applied to data generated from a 3D accelerometer attached to a smartphone. The algorithm employed three tiers—a pre-elimination tier to apply initial filtering, a double thresholding tier to detect harsh falls and physical activities occurring at a slow pace, and a machine learning tier to recognize slow falls and fall-like events using ML techniques. Energy saving was reported to be 62% compared with ML-only techniques, while the accuracy with the hybrid model was 93%. The hybrid approach was superior with respect to sensitivity and performed comparable to the threshold-based and ML-based approaches in terms of specificity.

There have also been various techniques to improve the performance of the algorithms used for fall detection, by optimizing preprocessing of data, influencing the feature selection/extraction, and applying ensembles to fall detection.

In [39], an example of a system that applies intelligent preprocessing to the data before applying machine learning for fall detection is given. In this, the authors apply a windowing technique to divide the sensor signals into windows or time segments. Classification algorithms were then applied to each window, to determine whether the activity in that window corresponded to a fall. In this, two Sun SPOT sensors attached to the chest and thigh were used, and it was observed that Naïve-Bayes classifier gave 100% accuracy and 87.5% sensitivity. Other algorithms used were SVM, OneR, C4.5 (J48), and neural networks. The objective of [40] was to distinguish falls from ADLs. In this study, the wearable fall detection system comprises a wearable motion sensor and a smartphone. The system works by analyzing not instantaneous values of acceleration and angular velocities, but by applying sliding windows to analyze streams of data. It applies Kalman filter to preprocess the raw data for noise reduction and Bayes network classifier for fall detection. The algorithm presented an ability to distinguish simulated falls from ADLs with an accuracy of 95.67%, sensitivity of 99.0%, and specificity of 95.0%. Zhao et al. [41] also apply a windowing technique to real-time data obtained from a triaxial gyroscope. The data were divided into a set of consecutive and partially overlapping windows. Three time domain features (resultant angle change, maximum resultant angular acceleration, and fluctuation frequency) were extracted from the data windows. Decision tree classifier was then used to classify each window as a fall or a nonfall event. The detection algorithm gave accuracy of 99.52%, precision of 99.3%, and recall of 99.5%. Another recent research [42] compares the performance of 4 algorithms—ANN, kNN, quadratic SVM, and ensemble bagged tree—in two steps. First, only acceleration and angular velocity data are used. Then, new features that improve the performance of the classifier are extracted from the power spectral density of the acceleration. The accuracy of the algorithms is observed to have increased after applying feature extraction techniques.

The objective of [43] was to test the impact of optimal feature selection on the accuracy of fall detection. The features of accelerations in different parts of the body are collected through wearable devices. Bayesian framework was applied to select the optimal features from the data generated by the wearable devices, and the weight of each feature was calculated, after which training was done based on the optimal feature set. It was observed that improved classification led to better accuracy, sensitivity, and specificity when compared to Naïve-Bayes and C4.5 classifications. Tsinganos and Skodras [44] analyze accelerometer data to extract a set of 14 features across time domain, statistical measures, and continuous wavelet transform. ENN was applied to remove outliers and then trained using kNN classifier to distinguish falls from ADLs. To negate individual-specific patterns, personalization was applied by appending the features of ADLs to the training dataset. The other models used for comparison were ANN, SVM, and J48 decision tree. The performance of kNN was the highest.

In [45], the authors propose EvenT-ML, in which a fall event was aligned with three stages of falls (preimpact, impact, and postimpact) using a finite state machine. The experimentation was based on data generated by accelerometers, and features were extracted from each phase. Classification and regression tree (CART), kNN, logistic regression (LR), and SVM were used to train the classifiers. The authors observe better results for EvenT-ML than the commonly used data segmentation techniques of fixed-size nonoverlapping sliding window (FNSW) or fixed-size overlapping sliding window (FOSW), where feature extraction is performed on all data segments. The finite state machine ensures that feature extraction gets executed only when the subject is in the active state, and this reduces the computational complexity of this method.

Recent research has also focused on the application of ensembles to fall detection. Hsieh et al. [46] use a combination of threshold-based and knowledge-based approach based on SVM, on data from a triaxial accelerometer, to detect a fall event. Absolute falls and ADLs are detected using thresholds on acceleration. In order to distinguish falls from ADLs in cases where the peak values of acceleration overlap, a knowledge-based approach is applied. Using this approach, sensitivity, specificity, precision, and accuracy were over 99%. Genoud et al. [47] propose a system for soft fall detection using ML in wearable devices. The feature sets used were linear acceleration and gyroscope readings, and the algorithms compared were decision tree, decision tree ensemble, kNN, and multilayer perceptrons (MLP). The experiments showed that decision tree ensemble outperformed the results obtained by the other algorithms. In [48], a comparison of Naïve-Bayes classifier, decision trees, random forests, random committee, and lazy learning (IBk) algorithms for activity detection was done. This used data generated by motion, acceleration, or inertial sensors embedded in a smartphone worn by the subjects. Naïve-Bayes classifier performed reasonably well for a large dataset, with 79% accuracy, and it was also the fastest in terms of building the model taking only 5.76 seconds. Random forest was better in terms of both accuracy and model building time, with 96.3% accuracy and 14.65 seconds model building time. k-Means clustering performed poorly with 60% classification accuracy and 582 seconds model building time. Kao et al. [49] use an ensemble of spectrum analysis, with GA-SVM, SVM, and C4.5 classifiers. The sensor readings were from 3-axis accelerometers. The best results were given by GA-SVM, with an accuracy of 94.1%, sensitivity of 94.6%, and specificity of 93.6%. Jahanjoo et al. [50] propose a fall detection algorithm based on data from 3-axis accelerometers, using PCA for dimension analysis and a multilevel fuzzy (MLF) min-max neural network, and compared the performance with MLP, kNN, and SVM. Using only 5 dimensions of features, MLF performed better than the other algorithms in terms of sensitivity, while the specificity was comparable for all four algorithms.

Hussain et al. [51] apply kNN, SVM, and Random Forest algorithms to not just detect falls, but also to identify the falling pattern and identify the activity that may have caused the fall. It is reported that the fall detection accuracy was highest for kNN, while the accuracy for recognizing different activities was highest for random forest. Yet another research [52] attempts to find a correlation between sampling rate and performance accuracy of machine learning models. In this paper, the authors compare the performance of SVM, Naive-Bayes, kNN, and decision trees with various sampling rates of sensors. It is concluded that with sampling rates of 11.6 Hz and 5.8 Hz, SVM and radial basis function gives accuracies of 98% and 97%, respectively. The research suggests that a sampling rate of 22 Hz is sufficient for most machine learning models to provide an accuracy of 97%.

Hakim et al. [53] propose a hybrid approach between threshold and ML-based fall detection algorithms. In this, a threshold-based algorithm is implemented to detect falls while a supervised machine learning algorithm is used to classify ADL. Data were collected from IMU sensors in a smartphone. Four different classification algorithms were used for detection and classification: SVM, decision trees, kNN, and discriminant analysis.

Our approach using SVM is also supported by the results which show that activity recognition can be increased with the accuracy level as high as 99%, when the combination of acceleration, angular velocity, and orientation parameters are utilized compared to using them separately.

The application of deep learning techniques for fall detection using wearable devices has been an area of recent interest. Musci et al. [54] describe an RNN model with LSTM blocks on data generated by 3D accelerometers for fall detection. The paper observes that though it is difficult to distinguish high dynamic activities from falls, the approach described achieves a better overall classification. Fakhrulddin et al. [55] apply CNN to streaming time series accelerometer data, collected from body sensor networks (BSN), for fall and nonfall situations. In [56], a method of applying CNNs with 3 convolutional layers on data generated by accelerometers is described. The activation function in each layer is rectified linear unit (ReLU). However, the study indicates that scarcity of public datasets based on accelerometer and gyroscope makes it challenging to develop deep learning solutions for this kind of data. Also, deep learning techniques require high computational processors, which may not be well suited to the constrained nature of wearable devices. Torti et al. [57] detail the implementation of RNN architectures for constrained embedded devices on a microcontroller unit (MCU), for fall detection with triaxial accelerometers. The work also provides an abstraction of formulas for memory, computing power, and power consumption for the embedding of a generic RNN architecture on an MCU.

Li et al. [58] describe fusion of data from a triaxial accelerometer, a micro-Doppler radar, and a depth camera. The paper analyses the impact of sensor fusion on the performance of classifiers. The classification accuracy attained by means of this fusion approach improves by 11.2% compared to radar-only use and by 16.9% compared to the accelerometer. It was also observed that fusing information from three sensors increases the classification accuracy to 86.9% with the quadratic-kernel SVM classifier, and up to 91.3% using an ensemble classifier. Some studies have applied sensor fusion in combination with deep learning techniques for fall detection. For example, Dawar and Kehtarnavaz [59] use CNN-based sensor fusion system to detect falls and ADLs. Signals from depth camera and wearable sensors (acceleration and angular velocity) are fed as inputs into separate CNNs. The algorithm then fuses the scores generated by these two CNNs to produce a classification. Zhou et al. [60] also describe an approach of using 2 CNNs for initial processing of two types of inputs and subsequent merging of the results of the two CNNs to produce the final detection results. In this, the inputs are obtained from radar signals to detect velocities, acceleration of human body parts, and images from optical camera. Other works along similar lines include references [61, 62].

Table 2 shows a snapshot of the recent research in the application of machine learning to fall detection using wearable systems. All experiments in the presented literature are based on analysis of public datasets or falls simulated under controlled environments. The table qualitatively compares different algorithms for fall detection and summarizes their performance parameters such as accuracy, sensitivity, and specificity where available.

## 6. Biological Risk Factors on Falls

The risk of fall exhibited by a subject could be influenced by multiple factors such as age, biological and physiological health profile, and environmental conditions. We did a survey on the factors that impact fall in the elderly. Existing literature shows that the risk of fall based on various factors is presented in the form of odds ratio, based on actual observations. OR is the ratio of the probability of an event of interest occurring to the probability of that event not occurring. It helps in estimating the relationship between two binary variables. Andrade [64] explains the significance of OR in the medical field, especially in cases where logistic regression analyses are applied, to find out the impact of a risk factor on an outcome variable.

Bird et al. [65] study the impact of decrease in postural stability over short time frame on fall rates and observes that fall rates increase when postural stability decreases, despite maintaining leg strength. Environmental factors also play a role in a subject's fall rate. In some of the experiments that we had performed, we observed that the accuracy of fall detection increased with the addition of health profile as a feature set. We present a survey on the biological risk factors on falls.

Current research indicates the correlation between a person's biological health and the risk of his/her falling. Kronfol [66] details results from comprehensive studies conducted on falls and cites causes of falls to be primarily environment-related, gait disorders, vertigo, drop attack, confusion, postural hypotension, and visual disorders. The study also specifies the odds ratio of various risk factors such as weakness, balance and gait deficits, mobility, and cognitive impairments, on the risk of falling. The impact of behavioural risk factors such as ADL characteristics and environment on falls is also explained. In [67], the authors performed experiments on 163 elderly men and women aged 60–95 years and found that history of falls, poor vision, use of multiple medications, chronic diseases, use of walking aids, vertigo, and balance problems were associated with falls among the elderly population living in long-term care homes. Graafmans et al. [68] constructed a risk profile for recurrent falls that included five risk factors: mobility impairment, dizziness upon standing, history of stroke, poor mental state, and postural hypotension and found out the probability of recurrent falls on people exhibiting one or more of these risk factors. In [69], a method based on performing chi-square tests to compare fall risk and overall injury risk with various demographic, behavioural, and health-related variables is proposed. Odds ratios for the association of each risk factor with the outcome were estimated using bivariate analyses and multivariate logistic regression models.

There have also been studies on risk factors of falls on subjects with specific conditions. For example, Stanmore et al. [70] observe that, in subjects with rheumatoid arthritis, risk factors include swollen joints, use of psychotropic medications and steroids, poor balance, and VAS fatigue score. This study did not uncover any relation between gender or age and risk of falling, which may be indicative of the fact that risks arising from rheumatoid arthritis override those specific to age or gender.

According to Li et al. [71], the major risk factors resulting in fall-related injuries are intrinsic and not situational or environment-related. Vertigo, weakness of the legs, and history of cancer were found to be risk factors. Cattelani et al. [72] propose a system for fall risk estimation in the elderly. This study leverages the already existing analysis of fall risk and deduces a fall probability for a subject based on statistical methods.

A study on the impact of gender on the probability of fall was inconclusive. Chang and Do [73] study the implications of gender on risk factors for falls among seniors. The factors having high impact in men were different from those in women. For example, in men, history of stroke presented an OR of 1.91, while in women, it was 1.51, and the OR for arthritis was 1.27 in men and 1.36 in women. The study highlights the differences between men and women in the associations between falls and various biological and medical factors. However, due to various limitations, further research is required to better understand these gender differences and their implications for risk assessment. Gale et al. [74] report that there are certain gender-specific risk factors, such as incontinence (OR = 1.48) and frailty (OR = 1.69) in women, and older age (OR = 1.02), high levels of depressive symptoms (OR = 1.33), and being unable to perform a standing balance test (OR = 3.32) in men. The authors suggest that although some homogeneity between the genders in the risk factors that were associated with falls were observed, gender should be taken into account in designing fall-prevention strategies because of the existence of several gender-specific risk factors.  Table 3 summarizes the various biological factors that impact a subject's probability of falling.

## 7. Challenges in the Design of Fall Detection Systems

From our analysis, an observation is that machine learning algorithms applied to various datasets in the literature survey produce varying degrees of accuracy. This indicates that the performance of the algorithms is dependent on various factors such as the type and placement of the sensors, the fall pattern, related thresholds if any, the characteristics of the dataset, and possibly the preprocessing that has been applied to it. Most literature on wearable sensor-based methods indicates that the performance of the algorithms varied with the position of the sensors. The thresholds are dependent on the subject's physical parameters, and hence, the performance of threshold-based methods depends on the customizations set according to features of the experimentation environments.

The lack of datasets that support research in this domain is also to be noted. Some research depends on public datasets—while these are useful to perform initial comparative studies, the fact that no more information than what is provided by the dataset would be available is a hindrance in proceeding further with research using such datasets. Most research has been done on datasets generated via experimentations. For obvious reasons, the experiments for simulating falls in all these cases were conducted in controlled environments, which may not reflect real-life situations accurately. Again, for obvious reasons, real datasets on fall patterns of the elderly are not available.

## 8. Conclusions and Future Directions

In this paper, we performed a brief comparison of fall detection systems that rely on environmental sensor-based, vision-based, and wearable sensor-based techniques. We then did a comprehensive survey of application of machine learning in wearable sensor-based FDSs. The survey was done by taking into account the type of sensors used, their positioning, the dataset used for analysis, the machine learning algorithms employed, and their performance summary results. We also presented a survey of biological factors affecting a person's probability of fall. Our findings indicate that wearable systems for fall detection have the advantage of being less intrusive, especially for elderly people, and ML techniques have the ability to detect falls to a reasonable accuracy level. However, a wearable system consisting of a device such as a wrist band alone is insufficient to meet the requirements of a comprehensive FDS.

From our study, we have observed that systems capable of generating alerts on detecting falls have been designed, but they fall short in the ability to activate adequate alerts, while minimizing the cost and power requirements. For example, there are vision-based systems which work based on videos/images captured by a camera. Such systems may be expensive and have higher power and bandwidth requirements. Also, parameters such as pulse, heart rate, temperature, SPO_2_, and electrodermal activity, which would increase the accuracy of the fall detection algorithm, are not taken into account in such systems because they rely solely on image processing mechanisms. Non-vision-based systems (e.g., wearable systems) detect falls, but they are decoupled from video/image inputs such as those generated by a camera. The flip side is that false positives and false negatives may tend to be ignored; for example, a false positive would trigger unnecessary action from the caregivers, and a false negative may not get the attention it deserves, leading to potentially dangerous situations. We observe that there is scope for developing systems that combine sensor readings with image inputs—for example, those that support activation of cameras to take pictures in the event of detection of a fall, to assist a remote caregiver.

The literature survey does not show any indication of having considered a person's biological parameters or health history into a fall detection algorithm. We observe that while there has been considerable research into finding out the correlation between a person's health profile and his probability of fall (as indicated by the odds ratio), not much has been explored in evaluating the impact of this odds ratio on the performance parameters of various ML algorithms for fall detection.

We also note that there is no integrated system that considers the fall risk to detect falls and generate alerts and camera-assisted observations. In addition, while the existing systems focus extensively on fall detection, there is scope for building an FDS that implements a closed-loop feedback—one that learns from a subject's fall patterns/history and change in physiological parameters at the time of fall and trains the fall detection algorithm based on these factors, to enable accurate profiling.

Our team is working on building a fall detection system that applies machine learning techniques for fall detection of the elderly. Our work on the comparison of the performance of various machine learning algorithms on a public dataset for fall detection is given in [63]. As next steps, we plan to work on data generated by a combination of IMU and vital signs sensors which are designed to be integrated into a wrist band. These wrist bands would be worn by elderly people staying in old-age care homes, where the end-to-end system is meant to be deployed. The system is also being designed to have the capability to generate alerts accurately and in a timely manner, in the event of an abnormality. The moderately mobile environment that the deployment pattern presents has an impact on the network design to support the end-to-end functionality.

## Figures and Tables

**Table 1:** Threshold-based systems for fall detection using wearable devices.

Reference	Year	Dataset used	Sensors used	Sensor placement	Methodology	Observed performance
[21]	2009	Generated from experiments	Accelerometer, gyroscope	Chest, thigh	Three-step algorithm based on activity intensity analysis, posture analysis, and transition analysis, based on signals reported by accelerometer and gyroscope	Sensitivity = 91% Specificity = 92%

[18]	2011	Generated from experiments	3D accelerometer	Not specified	Algorithm based on first differences and first derivatives of sum of accelerometer readings along *X*, *Y*, and *Z* directions	Algorithm is reliable, simple, and real time

[19]	2015	Generated from experiments	Accelerometer	Waist	Quaternion algorithm using sum acceleration and angle information	Better sensitivity and specificity than threshold-based algorithms

[20]	2015	Generated from experiments	Accelerometer, HRV sensor	Not specified	Analysis of signals from accelerometer for movement detection and HRV sensor for stress detection	Accuracy = 96% to 100% (depending on the type of movement)

[25]	2017	Generated from experiments	3-Axis accelerometer, 3-axis gyroscope, 3-axes magnetometer	Shoulder, waist, and foot	Threshold-based method, applied to acceleration and Euler's angle (yaw, pitch, and roll), run on a mobile phone	Accuracy = 100% Specificity = 91.1% (shoulder), 100% (waist), 78.5% (foot) Sensitivity = 100% (for all three placements)

[27]	2017	Generated from experiments	G-force sensor	Smartphone	3-Phase detection based on thresholds to identify falls and smartphone drops	Specificity = 72% when compared to the specificity of 31% with 2-phase threshold-based algorithm

[23]	2017	Generated from experiments	MEMS accelerometers, RF signals	Waist + network of fixed motes within the home	Signal analysis based on threshold-based methods	Not specified

**Table 2:** Machine learning-based systems for fall detection using wearable systems.

Reference	Year	Dataset used	Sensors/dataset used	Sensor placement (if wearable system)	Methodology	Observed performance
[30]	2011	UCI dataset	3-Axes accelerometer, 2-axis gyroscope	Chest, thigh	Comparison of ML algorithms for fall detection using single node and two nodes	Accuracy of classification = 99.8%, with 2 nodes—one on the waist and one on the knee Naïve-Bayes classifier gave best results

[34]	2012	Generated from experiments	Accelerometer	Mobile phone	Comparison of SVM, SMLR, Naive Bayes, decision trees, kNN, and regularized logistic regression for fall detection	Support vector machines and regularized logistic regression were able to identify a fall with 98% accuracy and classify the type of fall (trips, left lateral, slips, right lateral) with 99% accuracy. Naïve-Bayes reported least accuracy

[29]	2014	Generated from experiments	Accelerometer, gyroscope, magnetometer	6 different positions on the body	Comparison of k-NN classifier, LSM, SVM, BDM, DTW, and ANNs algorithms	k-NN classifier and LSM gave above 99% for sensitivity, specificity, and accuracy

[22]	2014	Generated from experiments	Accelerometer	Mobile phone	Accelerometer data from wearable sensors to generate alarms for falls, combined with context recognition using sensors in an apartment, for inferring regular ADLs, using Bayesian networks	Provides statistical information regarding the fall risk probability for a subject

[48]	2015	Publicly available activity recognition dataset	Accelerometer, gyroscope	Smartphone	Comparison of Naive Bayes classifier, decision trees, random forests, classifiers based on ensemble learning (random committee), and lazy learning (IBk) algorithms for activity detection	Naive Bayes classifier performs reasonably well for a large dataset, with 79% accuracy, and it is fastest in terms of building the model taking only.5.76 seconds Random forests are better in terms of both accuracy and model building time, with 96.3% accuracy and 14.65 seconds model building time. k-Means clustering performs poorly with 60% classification accuracy and 582 seconds model building time

[47]	2016	Generated from experiments	3-Axes accelerometer	Not specified	Comparison of decision tree, decision tree ensemble, kNN, neural networks, MLP algorithms for soft fall detection	Decision tree ensemble was able to detect soft falls at more than 0.9 AUC
[31]	2016	MobiFall dataset	Accelerometer, gyroscope	User's trouser pocket	Comparison of Naïve-Bayes, LSM, ANN, SVM, kNN algorithms for fall detection	k-NN, ANN, SVM had the best accuracy—results for kNN: Accuracy = 87.5 Sensitivity = 90.70 Specificity = 83.78

[26]	2016	Generated from experiments	3-Axis accelerometer	Smartwatch	Threshold-based analysis of acceleration	Accuracy = 96.01%

[40]	2017	Generated from experiments	Accelerometer, gyroscope	Vest	Kalman filter for noise reduction, sliding window, and Bayes network classifier for fall detection	With Kalman filter Accuracy = 95.67%, Sensitivity = 99.0% Specificity = 95.0%

[38]	2017	Generated from experiments	3D accelerometer	Smartphone	Combination of threshold-based and ML-based algorithms—K-Star, Naive Bayes, J48	Energy saving = 62% compared with ML-only techniques Sensitivity = 77% (thresholding only), 82% (ML only), 86% (hybrid) Specificity = 99.8% (thresholding only), 98% (ML only), 99.5% (hybrid) Accuracy = 88.4% (thresholding only), 90% (ML only), 92.75% (hybrid)

[46]	2017	Generated from experiments	3-Axes accelerometer	Waist	Combination of threshold-based and knowledge-based approach based on SVM to detect a fall event	Using a knowledge-based algorithm: Sensitivity = 99.79% Specificity = 98.74% Precision = 99.05% Accuracy = 99.33%

[49]	2017	Generated from experiments	3-Axes accelerometer	Smartwatch	Spectrum analysis, combined with GA-SVM, SVM, and C4.5 classifiers	GA-SVM gave best results with Accuracy = 94.1% Sensitivity = 94.6% Specificity = 93.6%

[50]	2017	MobiFall dataset	3-Axes accelerometer	Not specified	Comparison of multilevel fuzzy min-max neural network, MLP, KNN, SVM, PCA for fall detection	Multilevel fuzzy min-max neural network gave best results with Sensitivity = 97.29% Specificity = 98.70%

[37]	2017	FARSEEING dataset	3-Axes accelerometer	5 locations on the upper body - neck, chest, waist, right side, and left side	Sensor orientation calibration algorithm to resolve issues arising out of misplaced sensor locations and misaligned sensor orientations, HMM classifiers	Sensitivity = 99.2% (experimental dataset), 100% (real-world fall dataset)

[11]	2017	Generated from experiments	3-Axes accelerometer	Chest	LWT-based frequency domain analysis and SVM-based time domain analysis of RMS of acceleration	Accuracy = 100% Sensitivity = 100% Specificity = 100%
[32]	2017	Generated from experiments	3-Axis accelerometer, 3-axis gyroscope	Waist	Backpropagation neural network (BPNN) for fall detection	Accuracy = 98.182% Precision = 98.33% Sensitivity = 95.161% Specificity = 99.367%

[39]	2010	Generated from experiments	Accelerometer	Chest, thigh	Naïve-Bayes, SVM, OneR, C4.5 (J48), neural networks	Naïve-Bayes gave best results Accuracy = 100% Sensitivity = 87.5%

[43]	2016	Generated from experiments	Accelerometer	Different parts of the body	Bayesian framework for feature selection, Naïve-Bayes, C4.5	Better accuracy with improved classification than Naïve-Bayes and C4.5

[33]	2016	Generated from experiments	3D accelerometer	Chest	SVM, kNN, complex tree algorithms applied on data generated by accelerometers	Accuracy and precision of SVM were the highest Recall was highest for complex tree

[44]	2017	Generated from experiments	Accelerometer (MobiAct dataset)	Not applicable	ENN + kNN (where ENN was applied to remove outliers), ANN, SVM, and J48	For ENN + kNN: Sensitivity = 95.52% Specificity = 97.07% Precision = 91.83%

[41]	2018	Generated from experiments	Triaxial gyroscope	Waist	Decision tree	Accuracy = 99.52% Precision = 99.3% Recall = 99.5%

[45]	2018	Cogent dataset, SisFall dataset	3D accelerometer, 3D gyroscope-Cogent dataset Accelerometer, gyroscope-SisFall dataset	Chest, waist	Event-ML, classification and regression tree (CART), kNN, logistic regression, SVM	Better precision and F-scores with Event-ML than FOSW and FNSW-based approaches

[42]	2019	Public datasets	Accelerometer, gyroscope	Chest, thigh	ANN, kNN, QSVM, ensemble bagged tree (EBT)	Extraction of new features from acceleration and angular velocity improved the accuracy of all 4 classifiers. Accuracy of EBT was highest (97.7%)

[51]	2019	SisFall dataset	Accelerometer, gyroscope	Waist	kNN, SVM, random forest	Accuracy for fall detection was the highest for kNN (99.8%). Accuracy for recognizing fall activities was the highest for random forest (96.82%)

[52]	2018	SisFall dataset, generated from experiments	Accelerometer	Chest/thigh, waist	SVM, kNN, Naïve-Bayes, decision tree	Accuracy and sensitivity of SVM were the highest (97.6% and 98.3%, respectively) for both datasets

[63]	2018	UMA dataset	Accelerometer, gyroscope, magnetometer	Wrist, waist, chest, ankle	kNN, Naïve-Bayes, SVM, ANN, decision tree	Without risk categorization: 81% for decision tree With risk categorization: 85% for decision tree
[56]	2019	Public datasets	Accelerometer	Not specified	CNN-based models for feature extraction	Highest accuracy reported = 99.86%

[57]	2018	SisFall dataset-original and manually labelled	Accelerometer	Not specified	RNN	Highest accuracy reported for fall detection: 83.68% (before manual labelling), 98.33% (after manual labelling)

[36]	2018	Generated from experiments	Accelerometer, gyroscope, magnetometer	Near the waist	kNN	Accuracy = 99.4%

[16]	2018	Generated from experiments	Accelerometer	Waist	Decision tree	Accuracy = 91.67% Precision = 93.75%

[54]	2018	SisFall dataset	Accelerometer	Waist	RNN with LSTM	Highest accuracy (after hyperparameter optimization) = 97.16%

[53]	2017	Generated from experiments	Accelerometer, gyroscope, proximity sensor, compass	Right, left, and front pockets	SVM, decision tree, kNN, discriminant analysis	Highest accuracy = 99% for SVM

[59]	2018	Generated from experiments	Depth camera, accelerometer	Waist	CNN	Accuracy of fall detection = 100%

[55]	2017	Public datasets	Accelerometer	Not specified	CNN-based analysis on time series accelerometer data converted to images	Accuracy = 92.3%

[58]	2017	Generated from experiments	Accelerometer, radar, depth camera	Wrist	Ensemble subspace discriminant, linear discriminant, kNN, SVM	Overall accuracy of ensemble classifier was the highest, after fusion of radar, accelerometer, and camera = 91.3%. This is an improvement of 11.2% compared to radar-only and 16.9% compared to accelerometer-only results

[62]	2018	Generated from experiments	Accelerometer, gyroscope, magnetometer	Hip	SVM, random forest	Without sensor fusion: Accelerometer precision = 86.23% Accelerometer recall = 87.46% With sensor fusion: precision = 94.78%, recall = 94.37%, with random forest

**Table 3:** Biological risk factors on falls.

Reference	Year	Population demographics	Relevant parameters [odds ratio]
[69]	2012	Adults 65 years and older, with focus on adults 85 years and older	Activity limitation due to health problems [1.13]; use of assistive devices [2.18]; diabetes [1.2]; history of stroke [1.32]

[70]	2013	Adults 18–88 years with RA	Swollen or tender lower extremity joints [2.0]; history of stroke or Parkinson's disease [1.8]; history of ≥2 falls in previous 12 months [4.3]; symptoms of feeling dizzy or unsteady [1.8]

[66]	2007	Older adults	Weakness [8]; balance deficit [5]; gait deficit [5]; visual deficit [9]; mobility limitation [8]; cognitive impairment [5]; impaired functional status [4]; postural hypotension [5]

[67]	2016	Adults aged 60–95 years	Visual deficit [1.851]; chronic conditions [1.633]; vertigo [2.237]; imbalance [3.105]; fear of falling [3.227]; history of previous falls [5.661]; postural hypotension [0.804]; use of assistive devices [2.139]; hearing impairment [1.543]

[68]	1996	Adults 70 years or older living in homes for elderly	Mobility impairment [5.0]; dizziness upon standing [2.3]; history of stroke [3.4]; postural hypotension [2.0]; urinary incontinence [2.6]; use of walking aid [3.2]; visual deficit [1.7]; history of falls [3.5]

[71]	2016	Medical records of elderly hospitalized patients	Cancer [2.71]; vertigo [4.35]; weakness of lower legs [2.15]
